# Vitamin Effects in Primary Dysmenorrhea

**DOI:** 10.3390/life13061308

**Published:** 2023-06-01

**Authors:** Alkis Matsas, Athanasios Sachinidis, Malamatenia Lamprinou, Eleni Stamoula, Panagiotis Christopoulos

**Affiliations:** 1 Second Department of Obstetrics and Gynaecology, Medical School, “Aretaieion’’ University Hospital, National and Kapodistrian University of Athens, 115 27 Athens, Greece; 2 Fourth Department of Internal Medicine, Hippokration General Hospital of Thessaloniki, School of Medicine, Aristotle University of Thessaloniki, 541 24 Thessaloniki, Greece; 3 Laboratory of Clinical Pharmacology, School of Medicine, Aristotle University of Thessaloniki, 541 24 Thessaloniki, Greece; 4 Department of Biotechnology, Centre of Systems Biology, Biomedical Research Foundation of the Academy of Athens, 115 27 Athens, Greece

**Keywords:** dysmenorrhea, vitamins, vitamin D, vitamin E, vitamin K, vitamin B1

## Abstract

Background: Primary dysmenorrhea is considered to be one of the most common gynecological complaints, affecting women’s daily activities and social life. The severity of dysmenorrhea varies among women, and its management is of high importance for them. Given that non-steroidal anti-inflammatory drugs (NSAIDs), the established treatment for dysmenorrhea, are associated with many adverse events, alternative therapeutic options are under evaluation. Emerging evidence correlates management of dysmenorrhea with micronutrients, especially vitamins. Purpose: The aim of this narrative review is to highlight and provide evidence of the potential benefits of vitamins for the management of dysmenorrhea. Methods: The articles were searched on PubMed, Scopus and Google Scholar. The searching process was based on keywords, such as “primary dysmenorrhea”, “vitamins”, “supplementation”, “vitamin D”, “vitamin E” and others. Our search focused on data derived from clinical trials, published only during the last decade (older articles were excluded). Results: In this review, 13 clinical trials were investigated. Most of them supported the anti-inflammatory, antioxidant and analgesic properties of vitamins. Particularly, vitamins D and E revealed a desirable effect on dysmenorrhea relief Conclusion: Despite the scarcity and heterogeneity of related research, the studies indicate a role of vitamins for the management of primary dysmenorrhea, proposing that they should be considered as alternative therapeutic candidates for clinical use. Nevertheless, this correlation warrants further research.

## 1. Introduction

Dysmenorrhea is a common menstrual disorder prevailing among adolescent and young females [1]. The disorder actually refers to painful menstrual cramps, and it is characterized by lower abdominal pain during menstruation [2,3].

The severity of dysmenorrhea varies among women and usually improves after childbirth [4]. Approximately 10–15% of women complain about severe pain, resulting in a negative impact on their daily activities and absenteeism from school and work [5]. The overall prevalence of dysmenorrhea ranges between 50% and 90% in various populations and it is considered to be one of the major problems in women’s health [6]. It is noteworthy that the World Health Organization(WHO) refers to the disorder as the main cause of chronic pelvic pain [7].

Dysmenorrhea is categorized into two types: primary and secondary. Primary dysmenorrhea occurs in the absence oforganic pelvic diseases, whereas secondary is associated with specific disorders [8]. In primary dysmenorrhea, the pain appears just before or at the beginning of menstruation and lasts for 8–72 h, approximately [9]. Additionally, some dysmenorrhea-related symptoms are fatigue, headache, nausea, vomiting and diarrhea [10]. Although its etiology is not precisely elucidated, a suggested mechanism correlates primary dysmenorrhea with an increase in endometrial prostaglandins. Many studies reveal a significant rise in the levels of prostaglandins E2α (PGE2α), F2α (PGF2α) and leukotriene during menstruation, a fact that can serve as a cause for intense uterine muscle contractions and cramps [9,11,12]. Prostaglandins derive from arachidonic acid through the enzymatic action of cyclooxygenase and lipoxygenase. An increased activity of theses enzymes has been observed in women with dysmenorrhea; thus, nonsteroidal anti-inflammatory drugs (NSAIDs), which inhibit these enzymes, are utilized as a first-line treatment [13].

NSAIDs are an effective treatment in alleviating pain in primary dysmenorrhea; however, these drugs are associated with many gastrointestinal adverse events [14]. Thus, alternative therapeutic options, such as dietary supplements, could reduce the use of NSAIDs and their side effects.

Recent studies highlight a correlation between diminished levels of micronutrients and primary dysmenorrhea [15,16,17]. Women worldwide suffer from micronutrient deficiency, which seems to be related to hormonal changes and the menstrual cycle. Evidence has shown decreased serum levels of vitamin D in the luteal phase of the menstrual cycle, while vitamin D metabolites reduce levels of inflammatory cytokines and prostaglandins [18]. It has been proposed that menstrual cycle dysregulation is related to inflammatory mechanisms and oxidative stress; therefore, vitamins with anti-inflammatory and antioxidants properties, such as vitamin A and vitamin E, can modulate inflammation, oxidative stress and prostaglandin production [19,20]. In this narrative review, we outline the potential therapeutic properties of vitamins in primary dysmenorrhea.

## 2. Vitamin Effects in Dysmenorrhea Management

### 2.1. Effects of Vitamin D

**Figure 1:**
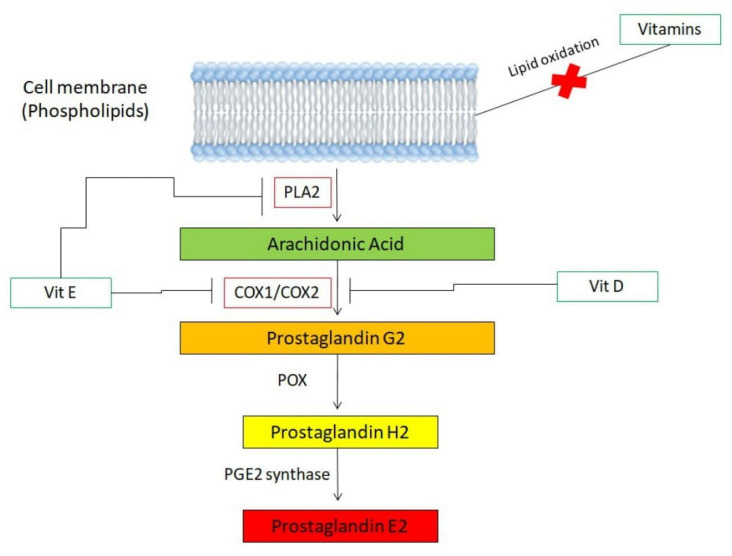
The enzyme phospholipase A2 (PLA2) releases arachidonic acid, a fatty acid, from the cell membrane. The acid transforms into prostaglandin E2 (PGE2), the primary prostaglandin responsible for dysmenorrhea, via cooperative functions of cyclooxygenases (COX1 or COX2), peroxidases (POX) and synthases. In the presence of Vit D, expression of COX2 is inhibited; thus, arachidonic acid is incapable of transforming into PGE2. Moreover, Vit E serves as an inhibitor of PLA2 and, similar to Vit D, suppresses expression of COX2 enzyme. Thus, arachidonic acid cannot be released from cell membrane and, if it is, its transformation into PGE2 is impossible. Of note, due to their antioxidant properties, vitamins inhibit the process of lipid oxidation, which leads to de novo synthesis of arachidonic acid.

Vitamin D (Vit D) plays a major role in the female reproductive system, as its receptors are present in ovarian and endometrial tissue, as well as in epithelial cells of the fallopian tubes, decidua and placenta [21]. Interestingly, it has been reported that Vit D deficiency correlates with severe to very severe dysmenorrhea [22]. Such a phenomenon can probably be explained by the fact that Vit D has anti-inflammatory properties via regulating prostaglandin levels [21,23,24]. In more detail, the biologically active form of Vit D suppresses cyclooxygenase 2 expression, thus reducing the prostaglandin production in the endometrium and affecting, as a result, calcium homeostasis (Figure 1) [21,23,24]. Of note, calcium intake has a protective effect on dysmenorrhea through controlling muscle nervous activities [25].

Taking into account all of the above, it is plausible to consider that Vit D supplementation—alone or in conjunction with calcium intake and/or other dietary supplements—may contribute to the relief of primary and/or secondary dysmenorrhea. Truly, a variety of studies suggest that high doses of Vit D can reduce the prevalence and also the severity of dysmenorrhea [1,26,27,28]. For instance, in Lasco et al.’s study [26], in which 40 women with dysmenorrhea were randomized, the treatment group received a single Vit D oral dose of 300.000IU, while the control group received the placebo. Over the two-month period of the study, significant pain reduction was observed in the Vit D group compared to the placebo [26]. In addition, in another study, by Moini et al. [27], a randomized control trial in 60 women with primary dysmenorrhea and Vit D deficiency was conducted. The treatment group received 50.000IU oral Vit D weekly for 8 weeks, while the control group received the placebo. Once again, two months after the beginning of the intervention, significant pain reduction was observed in the Vit D group compared to the placebo [27]. It is important to mention, however, that in a study conducted by Zangane et al., these beneficial effects of Vit D seemed to diminish in cases of moderate pain intensity [28].

Apart from the level of pain intensity, the time length of intervention seems to be another factor that strongly affects the benefits induced by Vit D supplementation [29]. Actually, a significant decrease in pain score between Vit D groups and placebo groups has been reported, but not before a two-month period after the beginning of the intervention [26,27,29]. According to a meta-analysis study, one month after the intervention, there seems to be no significant decrease in the pain score between the two groups (standardized mean difference −0.5, 95% CI: −1.6 to 0.6, *p* = 0.36) [29], whereas a month later (two-month period since the beginning), there is a significant decrease, as indicated by the statistics (standardized mean difference −1.02, 95% CI: −1.9 to −0.14, *p* = 0.024) [29].

In contrast to the data that recommend the use of Vit D to relieve dysmenorrhea [1,26,27,28,29], a study by Zarei et al. concluded that Vit D, combined with calcium, is incapable of decreasing pain severity [24]. Nevertheless, it is noted that the results of that study may be due to the absence of the Vit-D-alone group, as its assignment may have no further effect when calcium supplementation is sufficient [1,24].

In general, most of the available data indicate the possibility of using Vit D as a pain reliever in dysmenorrhea patients, as well as in other etiologies’ chronic pain cases [30]. The potential mechanism of Vit D action, which has been previously presented [29], supports such a fact. However, it is of high importance to make clear that clinical trials including larger participant groups are needed to assess the safety of these practices, as well as clarify the optimal level of Vit D in dysmenorrhea and its associated disorders [29].

### 2.2. Effects of Vitamin B1

Vitamin B1 (Vit B1), also known as thiamin, is a water-soluble vitamin with multiple functions. Actually, Vit B1 affects the muscle tone and neuronal activity of the body, as well as hematopoiesis and metabolism of carbohydrates [31]. Its use might also have a positive effect in primary dysmenorrhea symptoms, through womb muscular contraction and carbohydrate metabolism, that can alleviate mental and physical symptoms of the syndrome [31,32].

The effect of Vit B1 in primary dysmenorrhea was investigated in two clinical trials [29]. In the first study, 152 girls with severe and moderate dysmenorrhea were randomized [33]. The first group received 100 mg of Vit B1, while the second group received 400 mg of ibuprofen for a duration of two months [33]. The study results showed no significant difference in pain reduction between the two groups during the first month (*p* = 0.414), whereas a significant reduction was reported in the second and third months of the study (*p* < 0.01). Vit B1 use was further recommended because of a better safety profile [33]. In the second study, 240 girls with dysmenorrhea were divided into four groups. The first group received 100 mg of Vit B1, the second received 500 mg of fish oil, the third received both Vit B1 and fish oil, while the fourth received the placebo every day for a duration of two months [32]. At the end of the intervention, a significant reduction in pain severity was reported for all the intervention groups examined [32]. These data, in total, indicate that Vit B1, somehow, has positive effects on dysmenorrhea.

### 2.3. Effects of Vitamin K

An acupuncture point injection of vitamin K (Vit K) has been used as an effective treatment of dysmenorrhea, providing rapid pain relief and better quality of life [34,35]. Although the role of Vit K in the coagulation process is well documented, little is known about its role (and the underlying mechanism) on menstrual pain [35]. One clinical study has been conducted to assess the pain intensity and duration in dysmenorrhea patients treated with Vit K. The study also examined whether Vit K acupuncture point injection is an optimal treatment for patients [34]. In detail, 80 patients were eligible and were randomized in three groups [34]. The first group received an acupuncture point injection of Vit K3, the second group received a saline acupuncture point injection and the third received a deep K3 muscle injection [34]. The study results showed a significant reduction in the severity of dysmenorrhea in all three groups (in Vit K3 acupuncture point injection group, pain decreased from 8.0 to 1.5, *p* < 0.001, while in the saline injection group, decreased from 7.9 to 3.0, *p* < 0.001, and in the Vit K3 muscle injection group, decreased from 8.0 to 3.3, *p* < 0.001). The authors conclude that, according to the statistics, the acupuncture point injection of Vit K3 is considered as the optimal treatment, among those tested [34].

### 2.4. Effects of Vitamin E

Vitamin E (Vit E) displays an inhibitory role in the release of arachidonic acid and its conversion to prostaglandinvia action on the enzymes phospholipase A2 and cyclooxygenase (Figure 1) [36,37]. Due to these antioxidant properties, Vit E is considered as a potential dysmenorrhea reliever.

Truly, several studies conducted thus far confirm that Vit E supplementation is capable of alleviating dysmenorrhea and also reducing blood loss [38,39,40,41,42]. For instance, in a randomized double-blind placebo-controlled trial, 200 units of Vit E or placebo were given to the participants of the study (278 girls, aged 15–17 years old), and the severity and duration of pain, as well as the amount of menstrual blood loss, were measured at two to four months [41]. A visual analog scale was used for the pain record, whereas menstrual loss was measured via a Pictorial Blood Loss Assessment Chart (PBLAC) [41]. According to the results of the study, the Vit E group displayed lower pain severity, shorter pain duration and lower PBLAC score when compared with the placebo group, at both two and four months [41].

Another study suggests that Vit E supplementation is a good choice for dysmenorrhea patients that cannot use chemical drugs, such as ibuprofen [43]. It seems that ginger and/or Vit D serve as better choices for these patients, as they reduce the disease severity more effectively [44]. Interestingly, results from a meta-analysis study indicate that, as in the case of Vit D, Vit E’s positive effects on alleviating dysmenorrhea can only be reported at a period of at least two months into the intervention [29]. Thus, it is clear that some factors, such as the length of the intervention, strongly affect the effectiveness of Vit E in dysmenorrhea management and, due to that, more studies are needed in order to bring the maximum benefits of Vit E treatmentto patients.

### 2.5. Effects of Vitamin A and Vitamin C

Data regarding the effects of other vitamins in dysmenorrhea are extremely limited. One large study in adolescent girls was conducted, and the possible relationship between serum vitamin A (Vit A) status and inflammation status in subjects with primary dysmenorrhea and/or premenstrual syndrome was examined [45]. The results of that study suggest that serum Vit A, along with high-sensitivity C-reactive protein and prooxidant antioxidant balance, are significantly associated with the presence of premenstrual syndrome and primary dysmenorrhea [45]. Of note, Vit A (also known as retinol) is vital for sustaining multiple physiological actions, such as reproduction, morphogenesis and immune responses [19].

**Figure 2:**
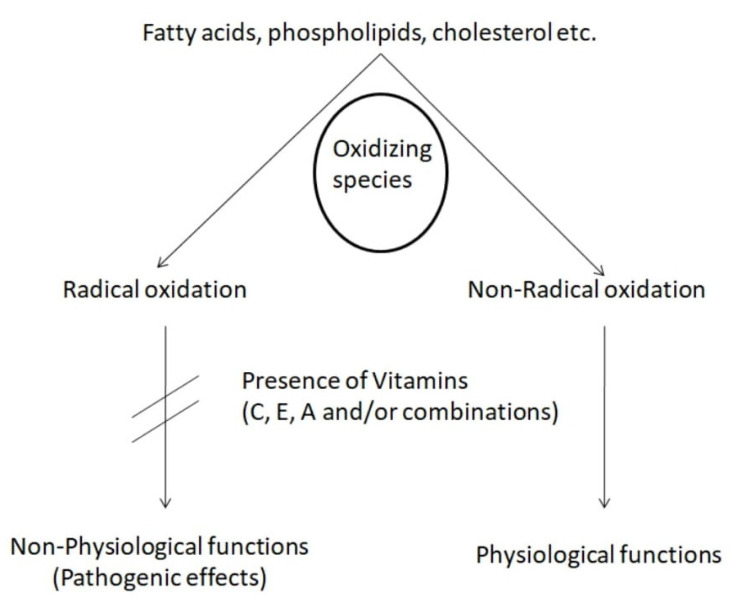
In the presence of Vitamin C, E and/or A, radical lipid peroxidation is inhibited. As a result, pathogenic effects are avoided. On the other hand, vitamins promote non-radical oxidation, which is associated with physiological functions.

As far as vitamin C (Vit C) is concerned, Venkata et al. reported reduced blood levels of the aforementioned vitamin in primary dysmenorrhea cases [46]. Vit C, also known as ascorbic acid, has the ability to remove oxygen-free radicals and, thus, plays a major role in the recycling of Vit E (via converting Vit Efree radical back to Vit E) to prevent fat peroxidation [47]. Interestingly, a randomized triple-blind placebo-controlled trial conducted in 2021 by Amini et al. suggested that the intake of Vit E and Vit C supplements has positive effects in women with endometriosis (whose symptoms include, among others, dysmenorrhea) [48]. In more detail, in that study, 60 reproductive women (aged 15–45 years old) were enrolled and randomized to two groups, one receiving a combination of Vit C and Vit E and the other receiving the placebo. The intervention lasted 8 weeks and, at the end of that period, a significant decrease in dysmenorrhea, pelvic pain severity and dyspareunia was reported in the treatment group, in comparison with the placebo [48]. Moreover, despite the fact that there was no significant decline in total antioxidant capacity after the treatment, the Vit E/Vit C group displayed a significant reduction in malondialdehyde and reactive oxygen species levels [48]. Taking into account these data, the authors concluded that Vit C, combined with Vit E, has an important role on the indices of oxidative stress and the severity of pain in women with endometriosis/dysmenorrhea (Figure 2) [48].

## 3. Pathways in Which Vitamins Alleviate Oxidative-Stress-Related Pain Modulation

Vitamins may reduce oxidative stress and, thus, modulate pain in an indirect manner. We present a few mechanistic pathways according to which vitamins E, C and D alleviate oxidative-stress-related pain modulation.

Vit E is an important protector of cell membranes from peroxidative stress; thus, it is believed to possess pain-alleviating actions [41]. Its analgesic properties may be attributed to the inhibition of the N-methyl-D-aspartate receptor (NMDA), in the dorsal horns of the spinal cord [49]. Of note, NMDA receptor antagonists seem to play an important role in pain relief [50]. Vit E has also been shown to activate theNuclear factor erythroid 2-related factor 2 (Nrf2) pathway, which leads to a reduction in oxidative stress [51]. NRF2 is a transcription factor that regulates redox homeostasis and, thus, orchestrates the defense of the cells against toxic and oxidative insults [52].

Vit C has been proposed to diminish markers of inflammation in blood circulation, probably via its antioxidant activity [53]. It is well established that pain sensitization is correlated with the release of inflammatory factors [54]. Thus, antioxidant and anti-inflammatory properties of this vitamin could contribute to pain alleviation. Interestingly, Vit C serves as a co-factor in the production of neuromodulators (such as serotonin, adrenaline and endorphins), therefore, exhibiting analgesic potential [55].

As far as Vit D is concerned, Nrf2 levels have been shown to be modulated by the aforementioned vitamin, as the oxidative stress activities are downregulated in its presence. Furthermore, Vit D supports redox control in cells by maintaining normal mitochondrial function and also facilitates the upregulation of antioxidant factors, anti-inflammatory cytokines and cell signaling, further regulating ROS [56].

Taking into account these data, it seems that vitamins’ antioxidant effects (as ROS scavengers, Nrf2 pathway activators and ant-inflammatory cytokines modulators) significantly reduce oxidative stress and, more importantly, cell injury and pain. Due to this, it can be strongly hypothesized that the use of vitamins in dysmenorrhea can not only reduce oxidative stress but also alleviate related pain. However, more studies are needed in order to elucidate the exact mechanisms of oxidative-stress-related pain modulation in dysmenorrhea.

## 4. In Vitro and In Vivo Investigations

Despite the fact that, in the review, emphasis has been put on data derived from clinical trials, we find it important to (also) comment on some interesting data that have emerged from in vitro and in vivo experimental procedures. These comments aim to demonstrate further connections between vitamins and primary dysmenorrhea, as well as to provide further research interests.

The effects of Vit D on PGE2 synthesis in osteoblast-like cells (MC3T3-E1 cell line) have been investigated [57]. According to the study, the active form of Vit D3 significantly inhibited the PGF2α-induced PGE2 synthesis in a dose-dependent manner. On the contrary, the pre-treatment of the cells with an inactive form of the aforementioned vitamin had little effect on prostaglandin synthesis [57]. Another study demonstrated that Vit D significantly reduced PGE2 production in human lung fibroblasts (HFL-1 cell line) and stimulated 15-hydroxy PG dehydrogenase, an enzyme that degrades PGE2 [58]. These findings, in total, suggest that Vit D can regulate PGE2 synthesis and degradation in vitro [57,58]. Furthermore, in human aortic endothelial cells (HAEC cells), Vit E increases the production of vasolidatorprostanoids through opposing the effects of the COX2 enzyme [59]. More specifically, Vit E increases the production of PGE2, PLA2 and arachidonic acid but inhibits COX production [59]. Taking into account all these data, derived from in vitro experiments, it is plausible to consider—even more, as data from clinical trials already reveal a desirable effect of vitamins on dysmenorrhea pain relief—that vitamins may serve as useful therapeutics for dysmenorrhea.

As far as in vivo models of the disorder are concerned, despite the fact that some mouse models of primary dysmenorrhea do exist [60,61], there are currently no reports regarding the in vivo effects of vitamins on dysmenorrhea. We note, however, that these animal models have been used in experimental procedures involving other supplements such as essential oils [62]. Interestingly, animal models of endometriosis have been investigated thoroughly, in terms of vitamin effects, revealing positive results in alleviating symptoms following the administration of Vit D [63]. Considering the fact that endometriosis is the most common cause of secondary dysmenorrhea [64], it can be strongly hypothesized that Vit D administration may also have beneficial effects on primary dysmenorrhea. In addition, in vivo studies involving mice macrophages showed that Vit E inhibits the release of COX and PLA2, thus inhibiting arachidonic acid metabolism [65,66]. These results also indicate that a similar mechanism may take place in the case of dysmenorrhea; however, further investigation is required regarding this issue.

## 5. Discussion

Primary dysmenorrhea is considered to beone of the main problems for women, affecting their quality of life and social activities [9]. Pain severity in primary dysmenorrhea is affected by many factors, and nutrient deficiency seems to be one of them [25]. Therefore, this narrative review focuses on elucidating the correlation between vitamins and primary dysmenorrhea and unraveling the potential mechanisms of their action.

The currently established utilization of NSAIDs raises many concerns, because of their multiple and severe adverse events, such as stomach ulcer, heartburn, gastrointestinal bleeding, hypertension, acute renal failure and worsening of pre-existing heart failure [14]. These adverse effects may be prevented by limiting NSAID dosage and duration or by seeking alternative therapeutic agents.

In spite of the paucity of clinical research, studies indicate the potential effects of vitamins on reducing pelvic pain severity. More specifically, it seems that vitamins D, B1, E, C and K (alone or in combination with other supplements) are capable of relieving dysmenorrhea (Table 1) [26,27,32,33,34,38,39,40,41,42,48]. The positive effects derived from vitamin supplementation usually require a two-month period into the intervention in order to be reported [29]. Of note, apart from the length of intervention, pain intensity is also a factor that affects the benefits induced [28].

Regarding the pathogenesis of dysmenorrhea, studies have attempted to shed light on the exact underlying mechanism. Excessive imbalanced amounts of prostanoids (prostaglandins, prostacylines, thromboxane A2) and eicosanoids (arachidonic acid), released from the endometrium, are thought to be implicated in the pathogenesis of primary dysmenorrhea. The abovementioned massive release of prostanoids and eicosanoids results in uterine hypercontractility, reduced uterine blood flow and nerve hypersensitivity, thus inducing dysmenorrhea [67]. NSAIDs alleviate pain through a mechanism involving the inhibition of the cyclooxygenase enzyme and the production of prostaglandins. Interestingly, many vitamins are believed to be involved in the molecular pathway of arachidonic acid, with vitamin D and vitamin E play a pivotal role [36,37,68,69].

Apart from the inhibition of enzymes in the arachidonic acid pathway, it is well established that vitamins possess antioxidant activity. Given that the arachidonic acid pathway is initiated viathe oxidation of membrane fatty acids, vitamins can limit arachidonic acid production and, thereby, its conversion to pain-inducing prostaglandins [70]. Vitamin E, due to its significant antioxidant effect, is the first line of defense against the peroxidation of membrane phospholipids. Vitamin C (ascorbic acid) is a free radical scavenger and exerts its antioxidant action through the conversion of vitaminEfree radical back to vitamin E [45]. Regarding the association between vitamin E and dysmenorrhea relief, studies support the pain-alleviating effect of vitamin E and itsanti-inflammatory action [40,44,71].

Before concluding, we consider it important to mention some limitations of our narrative review. The data described here refer to the last decade; thus, older results regarding vitamin supplementation in dysmenorrhea are excluded. However, we cannot ignore the fact that the effects of other vitamins, such as B3 (niacin) and P, werealso examined for their relieving abilities in dysmenorrhea in the 1950s [72,73]. Although we would like to make a comment regarding these vitamins in dysmenorrhea, we note that we didnot manage to reach that specific area of the literature via PubMed, Scopus and/or Google Scholar (only some reports, referring to the primary manuscript, are available on the internet). As far as vitamin A is concerned, we did not find a direct connection of the vitamin with dysmenorrhea and, as a result, no mechanism of action has been proposed. Still, we note that the antioxidant activity of vitamin A against lipid peroxidation has been reported in various studies [74,75,76]. Taking into account these studies, a scenario regarding vitamin A’s mechanism of action in dysmenorrhea may be considered as plausible (obviously, clarifications are required). Lastly, the majority of the studies described in our review were conducted in Middle Eastern countries [32,38,39,40,41,42,44,48]. We do believe that such a fact is due to the general culture of the people in these countries, as well as their diet. However, considering the fact that the results of all these studies indicate positive effects of vitamin supplementation in dysmenorrhea, it may be beneficial to consider vitamin supplementation as a therapeutic approach for dysmenorrhea for people of different ancestries (such as Europeans).

Taken together, this narrative review highlights a promising role of vitamins towards their potential therapeutic applications in primary dysmenorrhea. However, given the inconsistency and scarcity of studies and clinical trials, more studies are needed to confirm the safety, effectiveness and optimal dose of vitamins. Therefore, expanding our knowledge on the pathogenesis of dysmenorrhea and the multifaceted role of vitamins could optimize pain-relieving therapeutic strategies and maximize the benefits for women’s social lives.

## Figures and Tables

**Table 1:** Clinical trials conducted regarding the effects of vitamins D, K, B1, E and C in dysmenorrhea.

Author	Type of Study (Method)	Subjects	Intervention	Outcome
Lasco et al. [26]	Double- blind placebo-controlled randomized clinical trial	40 women 18–40 years old.	20 women received a single dose of vitamin D (300.000IU), while the control group (20 women) received the placebo.	There was a significant reduction in pain severity in the intervention group compared with the placebo.
Moini et al. [27]	Double -blind placebo- controlled randomized clinical trial	50 women with vitamin D deficiency, aged 18–30 years old.	25 women received 50.000IU οf vitamin D, once per week, while the control group (25 women) received the placebo.	The pain severity was deceased significantly in the intervention group after eight weeks of treatment, and after two months the use of NSAIDS was reduced as well.
Zarei et al. [24]	Randomized placebo- controlled trial	85 women 18–32 years old.	The first group received 1000 mg of calcium and 1500IU of vitamin D daily. The second group received 1000 mg of calcium daily, while the control group received the placebo.	There was a reduction in pain severity in the vitD/calcium group and the calcium alone group, while the difference was statistically significant only in the calcium alone group compared with the placebo.
Zangene et al. [28]	Double- blind placebo- controlled randomized clinical trial	54 women 18–30 years old.	The intervention group received a single dose of vitamin D(300.000IU) for three consecutive cycles, while the control group received the placebo.	The pain severity in the vitamin D group was decreased after two and three months of the treatment, while the use of NSAIDS also decreased compared with the placebo.
Wade et al. [34]	Double- blind placebo -controlled randomized clinical trial	80 women 14–25 years old.	The first group received a vitamin K3 acupuncture point injection, the second group received a saline acupuncture point injection (control group) and the third group received a vitamin K3 muscle injection.	There was a significant reduction in the severity of dysmenorrhea in all three groups, with greater reductions in the group receiving the vitamin K3 acupuncture point injection.
Hosseinlou et al. [32]	Double- blind placebo- controlled randomized clinical trial	240 girls 13–18 years old.	The first group received 100 mg of vitamin B1, the second took 500 mg of fish oil, the third received both vitamin B1 and fish oil, while the fourth took the placebo, every day for the duration of two months.	The study results showed that there was a significant reduction in pain severity for all treatment groups.
Zafari et al. [33]	Clinical trial	152 women 18–22 years old.	The first group received 100 mg of vitamin B1, while the second group received 400 mg of ibuprofen, for the duration of two months.	In the first month, there was no significant difference between the severity of pain in the two groups. However, there was a significant difference in the second and third months of the intervention.
Kashanian et al. [38]	Double- blind placebo- controlled randomized clinical trial	94 women 18–25 years old.	The intervention group received 400IU of vitamin E daily for seven days and two consecutive cycles, while the control group received the placebo.	There was a significant difference in the pain severity between the two groups, in the first and second months.
Moslemi et al. [39]	Single- blind placebo- controlled clinical trial	65 women with primary dysmenorrhea.	The first group received 100IU of vitamin E every six hours for three days and two consecutive cycles, the second group received 46 mg of fennel extract, while the third group received the placebo.	In the two treatment groups, the pain severity was decreased compared to the placebo. The fennel extract, however, displays the greater effect.
Ziaei et al. [40]	Randomized placebo- controlled trial	100 girls 16–18 years old.	50 girls received five 100IU capsules of vitamin E per day for five days, while the control group received the placebo.	The pain severity of the two groups was reduced after the intervention. Moreover, vitamin E displayed the greatest reduction.
Ziaei et al. [41]	Double- blind placebo -controlled randomized clinical trial	278 girls 15–17 years old.	The treatment group received 200IU of vitamin E twice a day for duration of five days, while the control group received the placebo.	There was a statistically significant reduction in pain severity in both groups at 2 and 4 months, but the reduction was significantly greater in vitamin E group compared to the placebo, as well as the pain duration and menstrual blood loss.
Nasehi et al. [43]	Double -blind cross -over study	68 girls with dysmenorrhea.	Two groups received, cross- over, a combination of vitamin E/fennel and ibuprofen for two months.	The combination of vitamin E/fennel extract is effective in decreasing pain intensity; thus, it can be used in patients where chemical drugs are not indicated.
Amini et al. [48]	Randomized triple-blind placebo-controlled clinical trial	60 women 15–45 years old.	The first group received 1000 mg/day of vitamin C, the second group took 800IU/day of vitamin E and the third group received the placebo.	After the 8 weeks period of the intervention, a significant decrease in pain severity was reported in the treatment group, in comparison to the placebo.
